# Social Interaction Improved by Oxytocin in the Subclass of Autism with Comorbid Intellectual Disabilities

**DOI:** 10.3390/diseases7010024

**Published:** 2019-02-22

**Authors:** Haruhiro Higashida, Toshio Munesue, Hirotaka Kosaka, Hidenori Yamasue, Shigeru Yokoyama, Mitsuru Kikuchi

**Affiliations:** 1 Research Center for Child Mental Development, Kanazawa University, Kanazawa 920-8640, Japan; munesue@med.kanazawa-u.ac.jp (T.M.); shigeruy@med.kanazawa-u.ac.jp (S.Y.); mitsuruk@med.kanazawa-u.ac.jp (M.K.); 2 Department of Neuropsychiatry, Graduate School of Medical Sciences and Research Center for Child Mental Development, University of Fukui, Eiheiji 910-1193, Japan; hirotaka@u-fukui.ac.jp; 3 Department of Psychiatry and Research Center for Child Mental Development, Hamamatsu University School of Medicine, Hamamatsu 431-3192, Japan; yamasue-tky@umin.ac.jp

**Keywords:** autism, oxytocin, subclasses, intellectual disability, epilepsy, randomized controlled trial

## Abstract

Approximately half of all autism spectrum disorder (ASD) individuals suffer from comorbid intellectual disabilities. Furthermore, the prevalence of epilepsy has been estimated to be 46% of patients with low intelligence quotient. It is important to investigate the therapeutic benefits and adverse effects of any recently developed drugs for this proportion of individuals with the so-called Kanner type of ASD. Therefore, we investigated the therapeutic and/or adverse effects of intranasal oxytocin (OT) administration, especially in adolescents and adults with ASD and comorbid intellectual disability and epilepsy, with regard to core symptoms of social deficits. We have already reported three randomized placebo-controlled trials (RCTs). However, we revisit results in our pilot studies from the view of comorbidity. Most of the intellectually disabled participants were found to be feasible participants of the RCT. We observed significantly more events regarded as reciprocal social interaction in the OT group compared with the placebo group. In the trial, no or little differences in adverse events were found between the OT and placebo arms, as found in some other reports. However, seizures were induced in three participants with medical history of epilepsy during or after OT treatment. In conclusion, we stress that behavioral changes in ASD patients with intellectual disabilities could be recognized not by the conventional measurements of ASD symptoms but by detailed evaluation of social interactions arising in daily-life situations.

## 1. Introduction

Autism spectrum disorder (ASD) is a neurodevelopmental condition with lifelong various phenotypes and a high prevalence, estimated at 0.62–2% [1,2,3]. The core symptoms of ASD have consisted of communication defects with social memory impairment and restrict interest and/or repetitive behavior [4,5,6].

A large number of studies investigating the theory of mind (ToM), which refers to the ability to attribute one’s own mind to others and oneself [7], have been conducted in individuals with ASD. As a result, impairment of ToM has been proven to be among the influential theories explaining social deficits in ASD [8,9,10]. Notably, considering that social deficits appear by 12 months of age in infants later diagnosed with ASD [11], these infants are not likely to be endowed with ToM, which is an essential requisite for participating in a human community. Thus, the clinical domain of social deficits expressed symbolically as ‘autism’ results in serious maladjustment in individuals with ASD in their daily lives [12], and the societal domain of social deficits, conceptualized as ToM impairments, addresses critically interdisciplinary issues, including social psychology [13] and philosophy [14].

In clinical settings, there are no effective treatments for the various symptoms of ASD, except for irritability, which can be alleviated by risperidone or aripiprazole [15,16]. Regarding social deficits, no psychosocial interventions or pharmacological treatments have been effective [17,18,19], although recent researches suggest the effectiveness of risperidone or centanafadine for social disabilities based on the Aberrant Behavior Checklist (ABC) subscale of social withdrawal [16,18,20,21].

Since the pioneering study of maternal behavior induced by centrally administered oxytocin (OT) in virgin rats [22], studies have been conducted to investigate how the evolutionarily conserved OT modulates reciprocal interactions among individuals in each species of vertebrates [23,24,25]. In humans, many studies have focused on social cognition and prosociality promoted by OT in interpersonal relationships in typically developing individuals [26,27,28,29]. The results of these studies have suggested that OT could benefit individuals with ASD due to its favorable effects associated with sociality [30,31,32,33]. The endogenous OT systems are believed to be involved in the pathogenesis of ASD, based on results indicating low levels of plasma OT [34], alterations in OT peptide forms [35], sexually dimorphic patterns of OT associated with anxiety [36], dysregulation of OT signaling pathways [37] and common polymorphisms of the *CD38* gene (*CD38*) [38,39,40,41] and OT receptor genes (*OTR*) [42,43] in individuals with ASD.

Exogenous OT is expected to be a promising candidate for the pharmacological treatment of ASD [26,29,31,44], and several clinical trials on short-term OT administration have been performed over the last decade [45,46,47,48]. A meta-analysis of this research suggested that OT has significant benefits compared with placebo in the treatment of ASD, with a moderate effect size (Cohen’s *d* = 0.57) [49]. Notably, social deficits may be relieved by OT administration [46,47,48,50,51,52,53].

We must consider the important subclass (Kanner type) of ASD individuals with low intelligence quotient (IQ) and epilepsy for benefits of new drugs, because these subjects have not been considered as targets of clinical trials owing to doubt of no tolerance to such trials. Here, we reviewed and compared recent randomized controlled clinical trials (RCTs) of OT, and whether or not the primary, secondary and exploratory outcome measures were favourable, especially for improvement of social behavioral impairments.

## 2. Chronic Treatment with OT in Randomized Controlled Trials

Currently, several clinical trials have been started to investigate whether intranasal OT is beneficial or not in ASD [32,54,55,56,57,58,59,60,61,62], while RCTs with OT as a long-term treatment are much less numerable (Table 1). In addition, here, we specially mentioned three RCTs conducted under the support of grant-in-aid from “Integrated research on neuropsychiatric disorders” carried out under the Strategic Research Program for Brain Sciences by the Ministry of Education, Culture, Sports, Science and Technology of Japan from 2011 to 2016 ([60,61,62], Table 1).

As listed in Table 1, the first RCT of repetitive intranasal application of OT was published by Anagnostou et al. [63]. While no favorable observations in the primary outcomes of social cognition and social function were observed, reading the mind in the eyes and low quality level of life (repetitive behavior) were improved. Dadds et al. reported that none of the social indicators are different between OT and placebo groups [64]. Guastella et al. reported that parent beliefs about treatment allocation were associated with an improved reported treatment response as assessed by parent or caregiver report [65], indicating caregivers’ effects. Watanabe et al. showed improvement in the reciprocity domain of the Autism Diagnostic Observation Schedule (ADOS) as a result of the main outcome [66]. Yatawara et al. reported that social interaction and behaviour was different in the OT group than the placebo group, rated by the caregivers in the caregiver-rated social responsiveness scale [67]. Very recently, Parker et al.’s OT treatment enhanced social abilities as measured by the trial’s primary outcome, the Social Responsiveness Scale [68]. These trials displayed that nasal spray was well tolerated in ASD patients, including young patients. Although no effect has been reported in half of the reports, there is an indication to support the potential of OT as an intervention for subjects with ASD to help improve social interaction deficits in the other half of the reports.

Kosaka et al. have shown efficacy of OT in young adults with high-functioning autism [61]. Clinical Global Impression—Improvement (CGI-I) scores in the high-dose group were significantly higher than in the placebo group. However, this effect was not observed in the low-dose group, nor if we include female participants in the calculation. We found that >21 IU per day OT was more effective than ≤21 IU per day. Interestingly, it was found that an SNP in OT receptors (rs6791619) predicted CGI-I scores for ≤21 IU per day OT treatment, suggesting that efficacy of long-term OT administration (12 weeks) in young men with high-functioning ASD depends on the OT dosage and genetic background of the OT receptor, which contributes to the effectiveness of OT treatment of ASD.

Very recently, in another clinical trial reported by Yamasue et al. [62], 106 high-functioning ASD individuals were randomly assigned to a six-week intranasal OT (48 IU/day) group or placebo group in four different university hospitals. OT reduced the primary endpoint in ADOS reciprocity. However, placebo also reduced the ADOS score, indicating the clear placebo effect. With respect to the secondary endpoints, OT reduced ADOS repetitive behavior and increased the duration of gaze fixation on socially relevant regions compared with placebo. The current large-scale trial conducted in multiple places suggests the possibility for OT to treat ASD repetitive behavior, but not the social domain. Next, we describe the RCT on ASD subjects with low IQ [60].

## 3. ASD with Comorbidities

Approximately half (56%) of ASD individuals suffer from comorbid intellectual disabilities [69]. In addition, epilepsy is likely found in 46% of ASD patients with low IQ [70]. Because these subjects have rarely been subjects in the many recent biological and clinical studies [46,47,48,49,71], we must consider this important proportion of individuals with the so-called Kanner type of ASD [5]. Therefore, we investigated the potential therapeutic and adverse effects of intranasal OT administration, especially in adolescents and adults with ASD and comorbid intellectual disability, with regard to core symptoms of social deficits. The results of our study have been published in 2016 by Munesue et al. [60]. The study was unique, because we needed to seek which measurements are applicable in such ASD individuals, because it is relatively difficult to obtain objective measurements in these patients using instruments such as functional magnetic resonance imaging. Realizing this difficulty, we finally reached the goal at which the behaviors of these subjects were analyzed using lists of real-life events that were obtained from their caregivers and an involved doctor, which were analyzed by a psychologist and sociologist who were not directly involved in OT treatment. Here, we summarized this exceptional clinical trial for the ASD subjects associated with intellectual disability.

## 4. RCT for Male ASD Subjects with Low IQ

We used computer-generated randomization to assign participants at a 1:1 ratio to intranasally administered OT (16 international units per day) or to a placebo for eight weeks before crossover, with the allocation concealed by centralized randomization.

All of the participants in our study were diagnosed as having ASD (autistic disorder according to Diagnostic and Statistical Manual of Mental Disorders, fourth edition, text revision (DSM-IV-TR [72]), with the mean age of approximately 22.5 years old (15–40 years) and IQs ranging from unmeasurable to 69 (unmeasurable, *n* = 12; ≤19, *n* = 5; 20–34, *n* = 4; 35–49, *n* = 6; 50–69, *n* = 3). Twelve (40%) participants had experienced autistic regression, and 15 (50%) exhibited catatonia-like symptoms. The behavioral phenotypes of their social impairments were classified as follows: aloof, 21; passive, 6; and active but odd, 3. None of the participants had histories of well-known medical conditions associated with ASD, such as tuberous sclerosis.

Seven participants (23.3%) suffered from comorbid stable epilepsy, except for one participant who suffered recurrent attacks once or twice per year. Twenty-two participants (73.3%) had received psychotropic medications (antipsychotics, *n* = 16; anticonvulsants, 12; hypnotics, 4; selective serotonin reuptake inhibitors, 1; anxiolytic, 1) with stable doses over the three weeks prior to randomization.

All of the participants went to schools for the handicapped or vocational facilities during the weekdays, except for one participant who resided in a facility for the disabled. Although 28 participants did not suffer from medically serious conditions, one participant (21 years old) had exhibited bipolar mood swings and was diagnosed as having a mood disorder after the age of 17 years old, and another participant was undergoing medical treatments for hypertension, diabetes mellitus and hyperuricemia. Twelve individuals who were first-degree relatives, aged 15 years old or older, of 10 participants (33.3%), had undergone psychiatric consultations due to mood disorders (nine individuals), anxiety disorders (one individual) and unknown diagnoses (two individuals).

The baseline values of all of the outcome measures, except for the Clinical Global Impression—Improvement scale (CGI-I) and real-life assessment of social interaction, were not significantly different between the OT-first and placebo-first groups.

## 5. Outcomes

There were significant main effects for time and order on the Clinician-Administered Rating Scale (CARS). However, we observed no main effect of treatment [60]. This result remained unchanged even when the severity of intellectual disabilities or the existence of autistic regression was incorporated as a covariate. There were no effects of treatment on the CGI-I, the ABC, the Interaction Rating Scale Advanced (IRSA) or plasma OT concentrations. Main effects for time and order were found on the CGI-I and ABC but not on the IRSA or plasma OT concentrations.

## 6. Real-Life Assessments of Social Interactions

We noticed changes in event rates, regarded as reciprocal social interaction based on a real-life assessment of social functioning, caused by administration of OT and the placebo. There were significant main effects at six weeks of treatment (*p* = 0.0025, Fisher’s exact test; Figure S1; [60]), showing that the increase in the rates of events regarded as reciprocal social interactions was significantly greater during OT treatment compared with placebo administration. Various episodes that had not been previously observed for the participants are listed in Table 2 [60].

For example, a mother reported the following: ”My son (40 years old with profound intellectual disabilities) pours hot Japanese tea in a teacup from a teapot for me upon my request. Usually, he leaves the teacup standing. The other day, however, he further handed it to me. That was his first behavioral change. I was very happy by this.” (Figure S2.)

These episodes may merely refer to unintentional observations at the doctor’s office or free statements of recollection by caregivers on behavior at home. However, ‘reciprocal social interaction’ refers to descriptions determined as indicating the existence of interpersonal exchanges between a participant and the medical doctor or caregivers. Most of the social interaction we picked up on was very small changes in behavior. The change was not the type of change in which one’s character was greatly altered to another personality. Initially, most of the parents worried if OT leads to a replaced personality. After eight weeks of OT treatment, participants who were treated with OT frequently displayed such social interaction. The percentage of subjects with interaction events was significantly higher than that in the placebo arm [60], as shown in Figure S1.

## 7. Assessment of Harm: Epilepsy

The mean values of treatment adherence ranged from 96.3% to 99.7% in the OT-first group and from 97.2% to 99.5% in the placebo-first group during the 16 weeks of the treatment phase, indicating that these subjects were tolerant to the RCT for about four months.

We did not observe any significant differences in aversive events between the two OT and placebo arms ([60]; see also Table 2 in Cai et al. [71]).

One participant experienced seizures repeatedly after the seventh week in the OT arm and discontinued the study immediately after crossover [60]. A second participant, who received anticonvulsant treatment for epilepsy diagnosed at 10 years of age, suffered a seizure after several years in the post-medication phase due to somewhat poor adherence. A third participant had repeated generalized tonic–chronic convulsions three and four months after the end of this study for the first time in his life, and he has since received pharmacological treatment that has yielded an effective response.

## 8. Further Consideration

We noticed improvement in behaviors regarded as reciprocal social interactions in the daily lives of the participants with comorbid intellectual disabilities and epilepsy. These results are consistent with recent two trials, in that they observed improvement in the caregiver-rated social responsiveness scale or social responsiveness scale. Though we intended to set these scales as the primary outcome, we did not use a defined way to pick up episodes to indicate reciprocal social interaction. We need a new design to measure episodes followed by scoring, or to use the already established caregiver’s scales [67,68], according to the current results for ASD patients with low IQ.

The positive correlations between the basal plasma OT concentrations and the baseline severity of aberrant behaviors in our study [60] were partly in agreement with the previous results of research suggesting that higher OT levels were correlated with more delayed development in individuals with ASD [35]. Interestingly, another study showed a significant correlation between baseline OT levels and the baseline ratings of the Yale-Brown Obsessive-Compulsive Scale in individuals with obsessive-compulsive disorder [73], which sometimes co-occurs with ASD [74]. Furthermore, the current study suggested that ASD individuals with lower plasma OT levels may show greater responses to OT compared with those individuals with higher OT levels.

Furthermore, it has been reported that the changes associated with OT treatment in plasma OT level were correlated with those in ADOS reciprocity [62]. The correlation between the changes associated with OT treatment in plasma OT level and those in ADOS reciprocity was significantly greater. It is suggested that an optimized strategy realizing elevation of plasma OT level to a sufficient level can induce a significant improvement in ASD social core symptoms compared with placebo.

No adverse effects of long-term OT administration have been shown in case reports, case series or randomized clinical trials conducted in adolescents and adults with ASD [31,64,75,76,77,78]. We also did not find significant differences in the rates of adverse events between the OT and placebo arms. However, the seizures experienced by three of the participants should be taken into consideration. Seizures invoked by OT have hitherto been confined to obstetric complications [79,80]. In rodent studies, there have been conflicting results associated with OT when tested as an anticompulsive or a proconvulsive drug [81,82]. Closer attention should be paid to ASD in future trials of OT, especially in patients with comorbid intellectual disabilities, because the prevalence of epilepsy has been estimated to be 46.0% in ASD individuals with a IQ < 50 [83].

## 9. Conclusions

It has been reported that levels of OT have a considerable variation in children, and that those with low levels of OT have fewer social skills, regardless of whether they suffer from ASD or not [68]. Therefore, it is expected that the administration of OT to autistic patients could improve their condition. The results of such studies have not always shown beneficial effects of OT (Table 1). We found that no conventional measurements could detect the possible effects of OT; however, an alternative means of describing interpersonal events observed in real-life situations [60] or doctors’ impressions (CGI; [61]) demonstrated favorable effects of OT, and baseline plasma OT levels predicted OT-induced behavioral improvements (Figure S3). Future studies require a new design to clarify the efficacy of OT under circumstances similar to those faced by individuals with ASD in their daily lives as members of society.

It is important, especially after a recent request from the NIH in the USA, that new studies should be planned with a more significant sample of autistic individuals. Future RCTs have to ascertain whether the effects of OT are independent of the mode of administration of the substance, with intranasal or sublingual sprays being common options. In this respect, very recently a report by Yamamoto et al. [84] showed that peripheral OT is transported into the brain by receptors for advanced glycation end-products in mice, suggesting that central OT has behavioral effects in humans after intranasal OT administration. This solves the long argument in this field of whether or not OT effects are mediated by OT binding to peripheral OT receptors that in turn influence brain activity, or directly by central OT receptors after recruitment of OT into the brain.

## Figures and Tables

**Table 1:** Effects of intranasal repetitive application of oxytocin on individuals with autism spectrum disorder (ASD) in randomized controlled trials.

Study	Drug	Age	IQ	Male/Female	Dose/day	Duration	Primary Outcomes	Effect	Other Outcomes
Anagnostou 2012	OT	33.2	99	9/1	48 IU	6 weeks	DANNA GCI-I RBS-R	No No No	Reading mind improved
Anagnostou 2012	PL	33.8	118	7/2					
Dadds 2014	OT	11.8		19/0	24 IU	4 days	Eye-contact Child verbal contact Social skills	No No No	
Dadds 2014	PL	10.7		19/0					
Guastella 2015	OT	13.9	80	26/0	18 or 24 IU	4 or 8 weeks	SRS CGI-I	No No	
Guastella 2015	PL	14.0	93	24/0					
Watanabe 2015	OL	35.1	109	9/0	48 IU	6 weeks	ADOS recip.	Improved	
Watanabe 2015	PL	29.3	101	9/0					
Yatawara 2015	OT	5.7	97	14/1	24 IU	5 weeks	SRS-P RBS-R	Improved No	
Yatawara 2015	PL	6.7	97	13/1					
Munesue 2016	OT	22.6	24.9	15/0	16 IU	8 weeks	CARS	No	Social interaction improved
Munesue 2016	PL	37.5	24.9	14/0					
Kosaka 2016	OT	23.1	99.2	15/2	16 IU	12 weeks	CGI-I IRSA	Improved No	
Kosaka 2016	OT	24.8	102.3	13/6	32 IU	12 weeks	CGI-I IRSA	Improved No	Dose- and SNP-dependen-tly improved
Kosaka 2016	PL	24.9	98.5	15/3					
Parker 2017	OT	9.4	65	13/1	24 IU	4 weeks	SRS	Improved	
Parker 2017	PL	8.1	67	14/0					
Yamasue 2019	OT	27.6	106	53/0	48 IU	6 weeks	ADOS	No	Repetitive behavior improved
Yamasue 2019	PL	26.3	108	53/0					

OT, oxytocin; PL, placebo; IU, international unit; DANNA, Diagnostic Analysis of Nonverbal Accuracy; CGI-I, Clinical Global Impression—Improvement; SRS, Caregiver-Rated Social Responsiveness; RBS-R, Repetitive Behavior Scale—Revised; ADOS, Autism Diagnostic Observation Schedule; SRS-P, preschool version of the Social Responsiveness Scale; CARS, Childhood Autism Rating Scale; SNP, single-nucleotide polymorphism; IRSA, Interaction Rating Scale Advanced.

**Table 2:** Examples of episodes regarded as reciprocal social interactions. The descriptions of participants’ behaviors in the oxytocin group were obtained from the play and interview sessions written in the medical chart, modified from the report by Munesue et al. [60].

**A. Verbal Communication**
**A-1.** His mother said the following: “My sister’s family lives nearby and they visit two or three times a month. When they visited, I felt that my son interrupted my conversations with my sister more often than before.”
**A-2.** “He joined in with the family conversation.”
**A-3.** “When my son was alone with me, he did not initiate conversation very often before, but recently he has done so somewhat more frequently.”
**A-4.** His mother said the following: “When I have picked up my son at the school, he recently has begun to speak, for example, that he had done his best today or that he had acted violently. Previously, he only spoke when I asked him.”
**B. Flexibility in Behavior**
**B-1.** His mother said the following: “Recently, my son has been less lethargic and did not wander around as much as that seen previously.”
**B-2.** “My son sometimes has drawn pictures of *Anpanman* (a cartoon character) as always at home. Recently, *Anpanman*’s expression has appeared to soften. He did not write very often in the past but has been writing a lot lately.”
**B-3.** “There was one thing that he always wanted to buy, *Donbei* (instant udon noodles), whenever we go to a convenience store. However, the other day, when we told him, ‘Stop buying that,’ he did so. We have not experienced anything like this before.”
**B-4.** “When we tell him to stop a given restricted behavior, he sometimes stops.”
**C. Sympathy**
**C-1.** “He cared for his young brother, when the brother came back during a holiday.”
**C-2.** “He handed me my tea cup after filling it with tea, when I asked him to pour tea.”
**C-3.** “He looked at us with a calm face for a long time.”
**D. Attitude toward Life**
**D-1.** “Recent entries in the correspondence book from the vocational aid center indicated that he seemed to be more motivated while at work.”
**E. Self-Harm**
**E-1.** “Self-injurious behaviors have lessened a little”

## Supplementary Materials

Supplementary materials are available online at https://www.mdpi.com/2079-9721/7/1/24/s1.
