# Supplementary material for: Social Interaction Improved by Oxytocin in the Subclass of Autism with Comorbid Intellectual Disabilities
Source: Diseases. 2019 Feb 22;7(1):24. doi: 10.3390/diseases7010024 (PMC6473850; doi:10.3390/diseases7010024)
Supplement: Supplementary file 1 [file diseases-07-00024-s001.pdf]

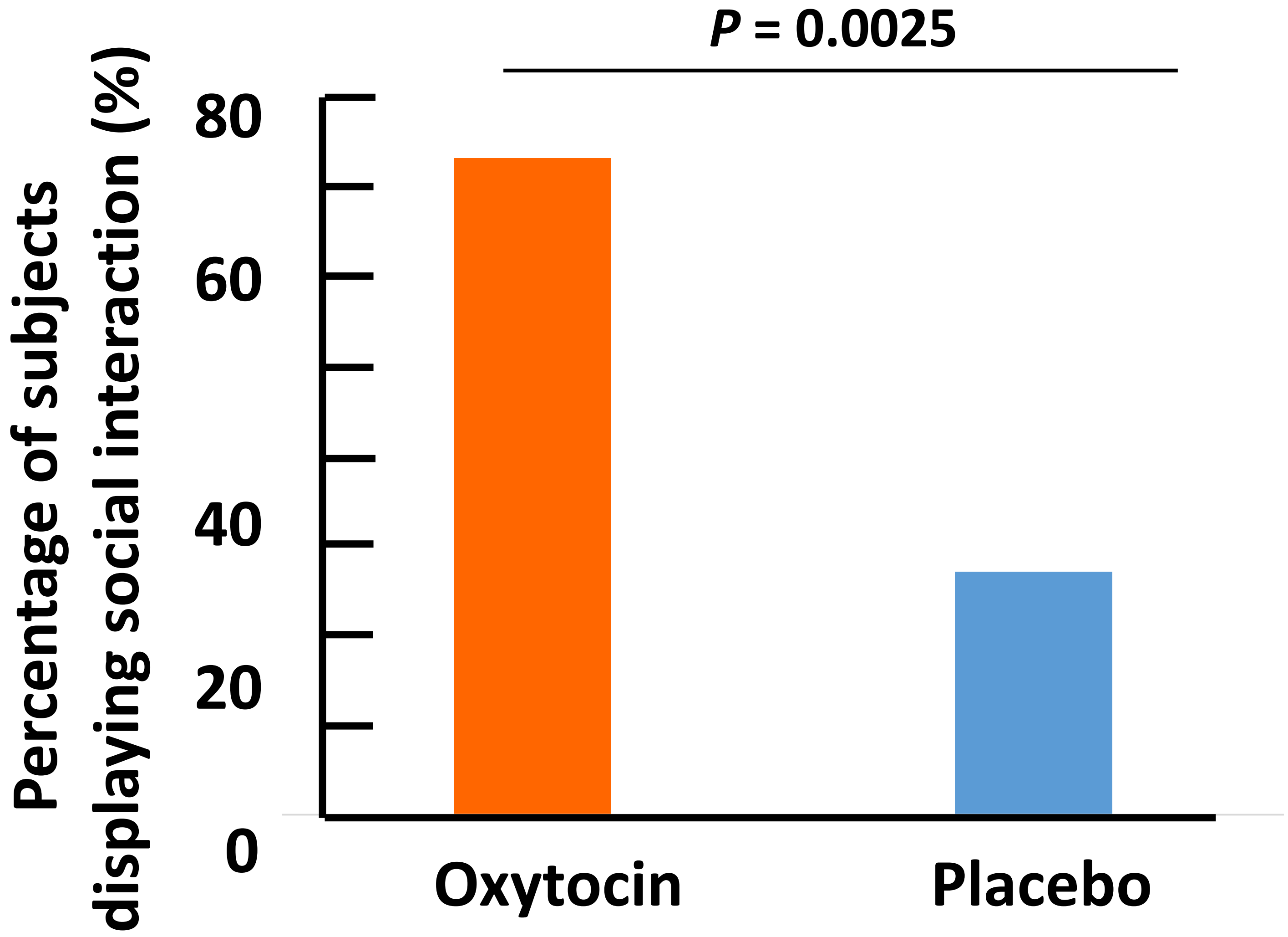

Supplementary Figure 1. Percentages of participants with at least one episode of social interaction. Percentages indicate the proportions of 29 participants who had at least one episode that was regarded as reciprocal social interactions at 6 weeks. Orange bar indicate the oxytocin (OXT)-first and blue bar represents the placebo first groups. Fisher's exact test, P = 0.0025. Data modified from Munesue et al. (2016).

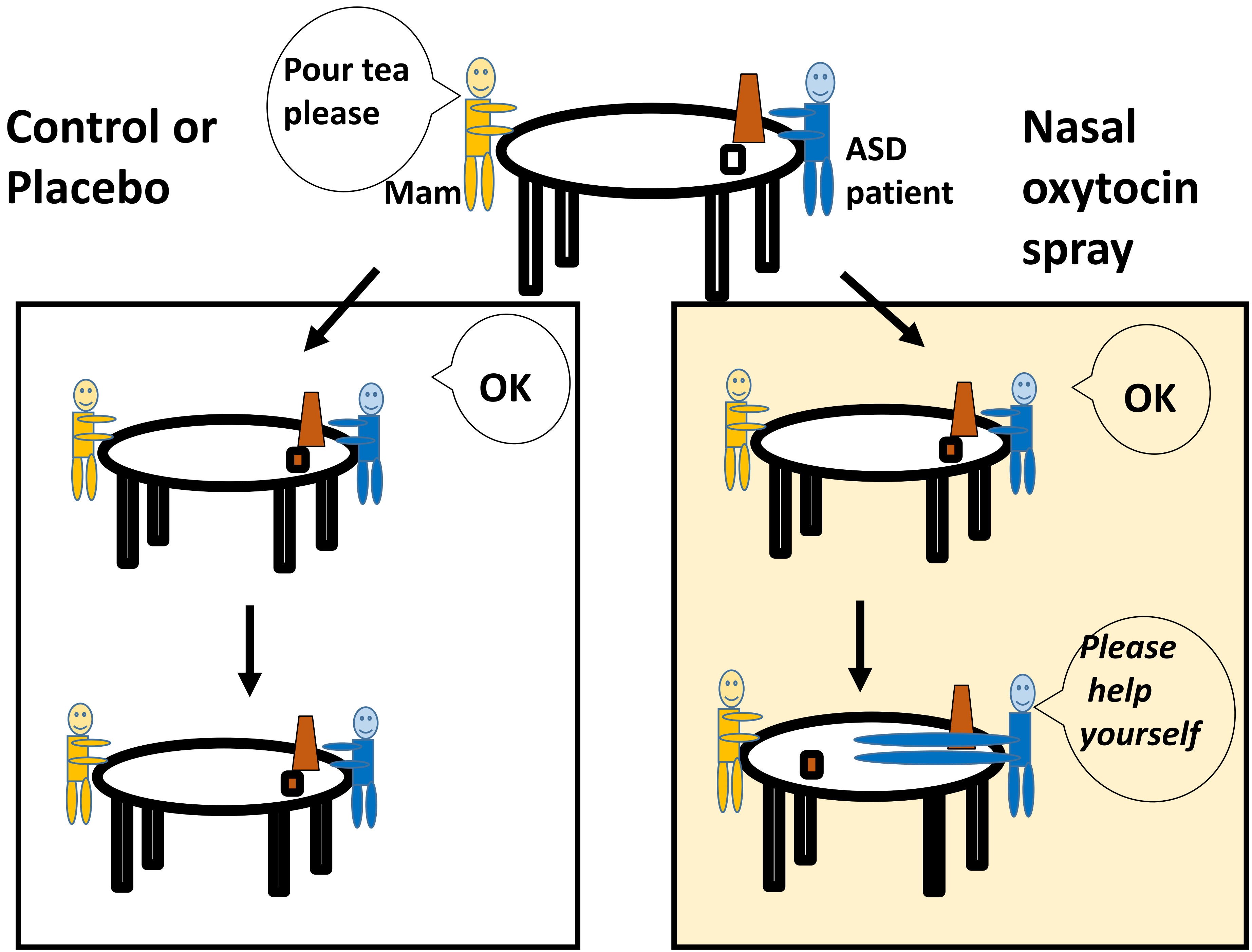
Control or Placebo
Pour tea please
Mam
ASD patient
Nasal oxytocin spray
OK
OK
Please help yourself

Supplementary Figure 2. Cartoon showing a typical case of mutual social interaction. A mother requested ‘to pour tea’ from a pot into her cup to her son of 40 years old with intellectual disability (Uppermost). He filled the cup with hot tea (middle). His reaction was stopped at this action always before OT treatment, because he completed his action according to his mother’s words (bottom left). He understood uni-directionally his mother’s request, and he never thought to pass the cup to her, because he could not understand her hidden desire that his mother wanted the tea-filled cup to drink for her purpose. But under OT treatment, he passed the cup to his mother, saying ‘Please, help yourself’ (bottom right). Now, treated with OT, the reciprocal interaction in response to his mother’s words occurred. His mother said this kind of interaction was never happen before, and it was the first time for her life to get a care from her son. Data based on the publication by Munesue et al. (2016)

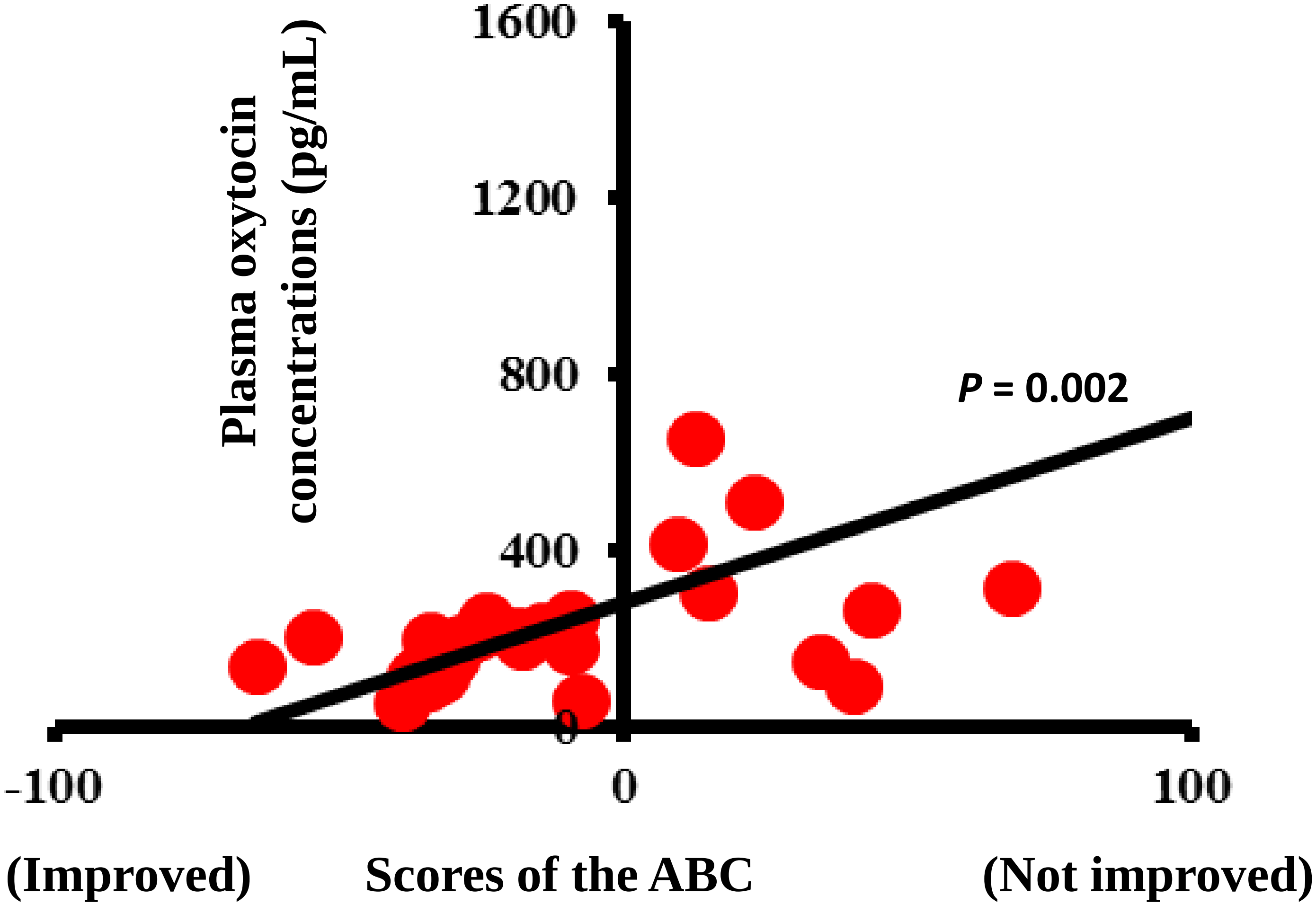

Supplementary Figure 3. Scatter plots portraying the relationships between plasma OXT concentrations and baseline scores on the Aberrant Behavior Checklist (ABC) after 6 weeks with OT treatment. The slopes of the dotted linear regression lines indicate a significant correlation (*P* = 0.002) in all of the participants. Data modified from Munesu et al. (2016).
